# Non-Cryogenic Structure and Dynamics of HIV-1 Integrase Catalytic Core Domain by X-ray Free-Electron Lasers

**DOI:** 10.3390/ijms20081943

**Published:** 2019-04-20

**Authors:** Jae-Hyun Park, Ji-Hye Yun, Yingchen Shi, Jeongmin Han, Xuanxuan Li, Zeyu Jin, Taehee Kim, Jaehyun Park, Sehan Park, Haiguang Liu, Weontae Lee

**Affiliations:** 1 Structural Biochemistry & Molecular Biophysics Laboratory, Department of Biochemistry, College of Life Science & Biotechnology, Yonsei University, Seoul 03722, Korea; jaehyun234@yonsei.ac.kr (J.-H.P.); jihye2@spin.yonsei.ac.kr (J.-H.Y.); jmhan@spin.yonsei.ac.kr (J.H.); zyjin@spin.yonsei.ac.kr (Z.J.); thkim@spin.yonsei.ac.kr (T.K.); 2 Complex Systems Division, Beijing Computational Science Research Center, Beijing 100193, China; shiyc12@csrc.ac.cn (Y.S.); lixx11@csrc.ac.cn (X.L.); 3 Department of Engineering Physics, Tsinghua University, Beijing 100084, China; 4 Pohang Accelerator Laboratory, Pohang 37673, Korea; jaehyun.park@postech.ac.kr (J.P.); sehan@postech.ac.kr (S.P.)

**Keywords:** non-cryogenic structure, HIV-1 integrase, protein dynamics, XFELs

## Abstract

HIV-1 integrase (HIV-1 IN) is an enzyme produced by the HIV-1 virus that integrates genetic material of the virus into the DNA of infected human cells. HIV-1 IN acts as a key component of the Retroviral Pre-Integration Complex (PIC). Protein dynamics could play an important role during the catalysis of HIV-1 IN; however, this process has not yet been fully elucidated. X-ray free electron laser (XFEL) together with nuclear magnetic resonance (NMR) could provide information regarding the dynamics during this catalysis reaction. Here, we report the non-cryogenic crystal structure of HIV-1 IN catalytic core domain at 2.5 Å using microcrystals in XFELs. Compared to the cryogenic structure at 2.1 Å using conventional synchrotron crystallography, there was a good agreement between the two structures, except for a catalytic triad formed by Asp64, Asp116, and Glu152 (DDE) and the lens epithelium-derived growth factor binding sites. The helix III region of the 140–153 residues near the active site and the DDE triad show a higher dynamic profile in the non-cryogenic structure, which is comparable to dynamics data obtained from NMR spectroscopy in solution state.

## 1. Introduction

The study of protein dynamics has progressively improved with the discovery of experimental techniques such as hydrogen deuterium exchange mass spectrometry, molecular dynamics (MD) simulation, and solution nuclear magnetic resonance (NMR). However, the methods that allow for the analysis of the structure and the dynamics of the protein simultaneously are limited to MD simulation and solution NMR. In addition, these techniques have limitations such as the size of molecules or the experimental support needed to validate the simulation results. In recent years, the development of serial femtosecond crystallography (SFX) using X-ray free electron lasers (XFELs) at room temperature has allowed for the dynamics and structural enzymology of proteins [1,2]. Mix-and-inject serial crystallography (MISC) was designed to image enzyme catalyzed reactions in which small protein crystals are mixed with a substrate [3,4,5]. However, MISC is difficult, which limits its use in experiments. Therefore, although less reaction information is obtained compared to MISC, determining the protein thermal dynamics by analyzing both cryogenic and non-cryogenic structures is an efficient method in order to improve our understanding of dynamic protein behavior, especially regarding motions near the active site of enzymes at room temperature.

The integration of the viral genome into the host genome during the life cycle of the HIV-1 virus is an essential process of infection [6]. HIV-1 integrase (HIV-1 IN) plays a key role in virus integration without the need for any other co-factors [7,8]. HIV-1 IN catalyzes two reactions. The first is 3′-processing, in which two nucleotides are removed from one or both 3′-ends of the viral DNA to expose the invariant CA dinucleotides at both 3′-ends of the viral DNA [9,10,11]. The second reaction is the strand transfer reaction, in which the processed 3′-ends of the viral DNA are covalently ligated to the host chromosomal DNA [12,13]. HIV-1 IN is considered a good target for the development of therapeutic agents for the treatment of HIV-1 virus. Currently, raltegravir and elvitegravir, developed for targeting HIV-1 IN, have already been commercially successful, and new therapeutic agents targeting IN are under development [14,15,16]. 

HIV-1 IN consists of three domains: N-terminus, catalytic core domain, and C-terminus. The HIV-1 IN catalytic core domain (IN-CCD) itself has integration activity and the activity is eliminated when DDE triad (formed by Asp64, Asp116, and Glu152) located on this domain is mutated [14,15,16]. Neither the N- nor C-terminal directly affects the activity, however, the entire genome transfer process requires these three domains to cooperate. Structural studies of the HIV-1 IN have been reported using X-ray crystallography and cryo-electron microscopy [17,18,19,20,21,22,23,24,25,26]. However, these key regions which are important for the activity are often disordered due to a high flexibility and crystal packing. 

Since the dynamic properties of proteins at ambient temperature are highly related to their function, the structure of SFX by XFELs (SFX-XFELs) at non-cryogenic temperature showing its dynamics is very beneficial to determining the relationship between structure and original function. The combination of SFX and NMR could reveal more details in protein dynamics as well as structural changes in the active site.

To our knowledge, this is the first report of the non-cryogenic crystal structure of IN-CCD included their original dynamicity using SFX-XFELs. We analyzed the differences between the cryogenic and non-cryogenic structures. Although both structures were in overall agreement, the helix III and side chain orientations were found to be different. Interestingly, the electrostatic network of the active site of IN-CCD was dramatically different. In conclusion, this study provides an insight into the structural and thermodynamic differences of protein between at non-cryogenic and cryogenic temperatures, and the results encourage the application of SFX for further structural enzymology studies.

## 2. Results

### 2.1. Non-Cryogenic and Cryogenic Structures

**Figure 1:**
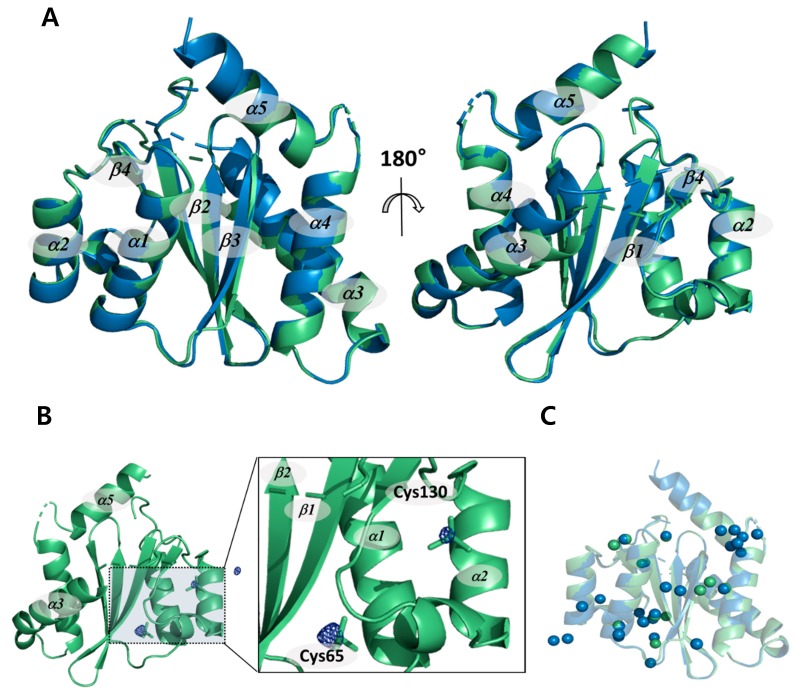
Comparison of the crystal structures of HIV-1 integrase catalytic core domain at both cryogenic and non-cryogenic temperature. (**A**) Superimposed non-cryogenic (green) and cryogenic (blue) structures. (**B**) Non-cryogenic (green) structure plotted together with the |F_obs_|^non-cryo^–|F_obs_|^cryo^ difference Fourier electron density map contoured level at 4.5 σ. Positive maps were marked by blue mesh. (**C**) Water molecule positions of non-cryogenic (green) and cryogenic (blue) structures.

Both the cryogenic and non-cryogenic structures of IN-CCD were solved by conventional synchrotron and SFX-XFELs (Table 1). Two structures share molecular topologies, with no significant differences. The root mean square deviation (RMSD) between the two structures is 0.31 Å, as shown in Figure 1A. In the |F_obs_|^non-cryo^–|F_obs_|^cryo^ difference Fourier electron density map contoured at 4 σ for the whole protein, there were very few positive and negative peaks in the backbone atom and the side chain atom. In contrast, we found very large positive peaks in the cacodyl ion region covalently linked to Cys65 and Cys130 (Figure 1B). This could be a feature of reduced radiation damage by SFX-XFELs with ultrashort pulses that allow measurements outrun X-ray damages. Normally, molecules such as cacodyl ions are susceptible to radiation damage, resulting in a diffuse electron density. In non-cryogenic structure from SFX-XFELs, there is no sign of radiation damage, such that the reduction in electron density caused by a diffuse electron density appears as a peak in the difference Fourier electron density map. The positive peaks located in cacodyl ions are evidence that the non-cryogenic structure did not undergo any radiation damage. Thus, unlike the synchrotron structure, which suffered radiation damage even at cryogenic temperatures, the non-cryogenic structure from SFX-XFELs shows exactly the expected result as the “diffraction before destruction” principle predicted [27].

In addition, the number of water molecules in the non-cryogenic structure is lower than the cryogenic structure, from 29 to 12 molecules (Figure 1C). This may be due to the relatively poor resolution of 2.5 Å compared with the cryogenic structure [28]. However, this phenomenon is thought to be a combination of poor resolution and changes in the dynamics of the water molecules as a result of temperature, since several water molecules that cannot be seen in the cryogenic structure are clearly visible in the non-cryogenic structure. If the problem were due to resolution only, this would not happen. In particular, the hydrogen bond network mediated by the water molecules around the Asn120 located near the active site changed. Since water molecules are used for nucleophilic attack in the 3′-processing reaction, these changes in the water molecules located near the active site may affect integrase (IN) catalysis [29,30,31]. This suggests that the changes in enzymatic activity due to temperature are related to this series of structural changes.

### 2.2. Structural Differences upon Temperature Difference

**Figure 2:**
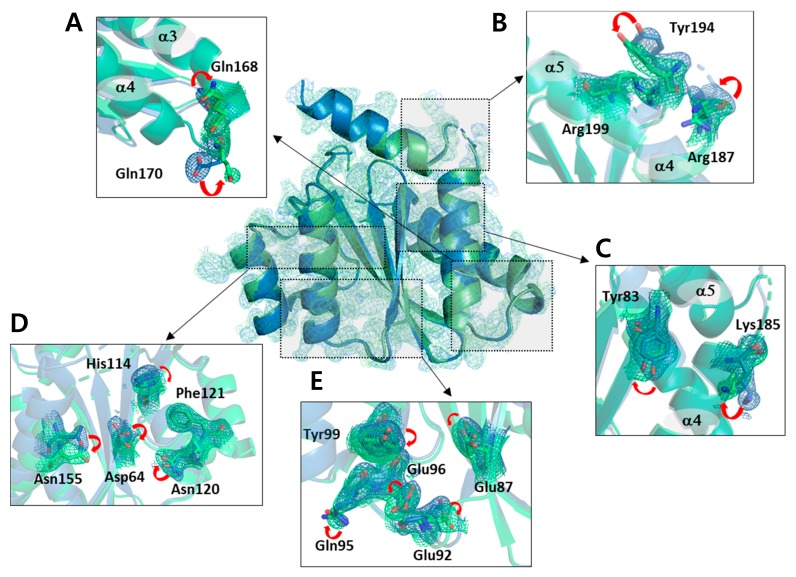
Orientation of the side chains observed at both non-cryogenic and cryogenic structures. The omit maps of side chain are presented at contour level 1.0 σ. The changes of sidechains located in **(A)** helix III-helix IV loop, (**B**) helix IV-helix V loop, (**C**) the end of beta sheet III and helix IV, (**D**) beta sheet IV and helix II, and (**E**) helix I and beta sheet III.

The structural differences found in the backbone atoms between the non-cryogenic and cryogenic structures were not significant. However, important changes in the side chain atoms were observed in several regions. Most of these changes corresponded to a shift in rotamer conformation and mainly occurred at the outer loop site. This region was found to be easily affected by thermodynamic changes in the atoms depending on the ambient temperature. However, not only the residues of the exposed part, but also the residues inside the protein changed greatly. In particular, we observed many of the side chain atom changes in the residues with relatively larger side chains such as tyrosine, lysine, arginine, histidine, glutamic acid, glutamine, aspartic acid, asparagine, and phenylalanine (Figure 2, Table S1). Since these residues have varying rotamer conformations compared to other residues, they are more likely to be involved in molecular interactions and thus have an effect on the overall dynamics.

The 167–174 residues located in the loop between helix III and helix IV make up the interaction site for the integrase binding domain (IBD) of the lens epithelium-derived growth factor (LEDGF), the binding partner of the IN [32]. In particular, the Asp167, Gln168, Glu170, His171, and Trp174 residues form hydrogen bonds with the IBD of LEDGF, which is important for the binding of IBD and IN. On comparison of the non-cryogenic and cryogenic structures, the Gln168 and Glu170 residues included IBD binding site showed important changes (Figure 2A). In previous studies, the x-ray crystal structure of IN-CCD/IBD has been reported [33], and IBD binding site of the non-cryogenic structure of the IN-CCD was in good agreement with that of the previous reported IN-CCD/IBD complex. This result indicates that the interaction mode of IBD and IN-CCD can vary depending on temperature. Changes in the side chain were also observed in the helix V elongated to the C-terminus and in the loop region around helix V (Figure 2B). Tyr83 was located in beta sheet III and Lys185 mutated from Phe to Lys to improve solubility and stability (Figure 2C) [34]. The structural changes found in this region suggest a thermal dynamic change due to temperature differences regardless of the function of the protein. Figure 2D shows the changes of residues His114, Asn120, and Phe121, as well Asn155 and Asp64, in beta sheet IV and helix II. The residues at beta sheet IV and helix II are known to be involved in the interaction with template DNA (tDNA) when the HIV-1 strand transfer complex (STC) intasome is formed [23]. In particular, Asn120 is known to form an electrostatic protein-DNA interaction with tDNA. Asn120 is connected to Asp64, one of the constituent residues of the DDE triad, via a water molecule. Due to the structural change of Asn120, the network mediated by surrounding water molecules also changed. Changes in the residues located in helix I and beta sheet III, the homo-dimeric interfaces of the IN-CCD, were observed, including Glu87, Glu92, Gln95, Glu96, and Tyr99. This region of the crystal structure is exposed to solvent, suggesting that these changes occur due to changes in ambient temperature, affecting thermal motion of the residues (Figure 2E). 

**Figure 3:**
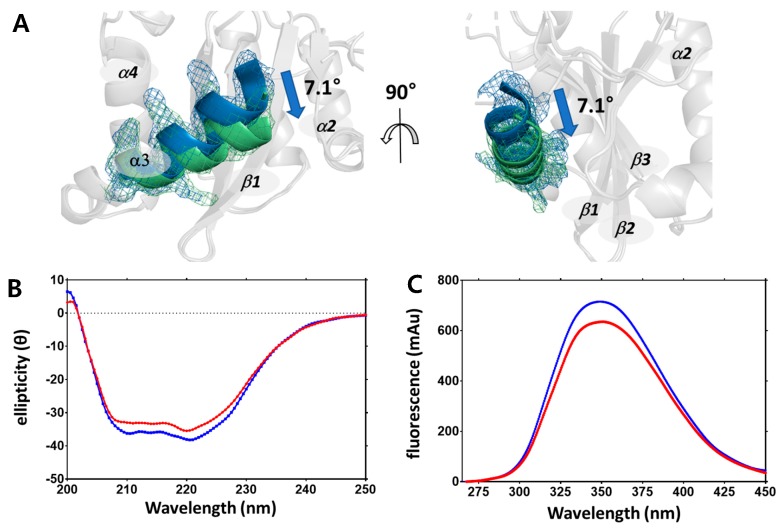
Changes in secondary structure presented by omit maps and circular dichroism and fluorescence absorbance. (**A**) Superimposed helix III region of non-cryogenic (green) and cryogenic (blue) structures plotted using an omit map at contour level 1.5 σ. (**B**) Circular dichroism spectra of HIV-1 integrase catalytic core domain at 4 °C (blue) and 25 °C (red). (**C**) Fluorescence intensity absorbance of HIV-1 integrase catalytic core domain at 4 °C (blue) and 25 °C (red).

Structural changes due to changes in temperature were not only found in the side chains but also in the secondary structure. When the cryogenic and non-cryogenic structures were compared, the orientation of the helix III was found to have shifted toward helix VI by 7.1 degrees (Figure 3A). Helix III plays an important role in IN activity in relation to the DDE triad, whereby a change in its orientation by 7.1 degrees alters the electrostatic interaction of the overall DDE triad. In addition, the lysine residues in helix III are important for insertion into the minor grooves of viral DNA (vDNA), but were difficult to compare due to many of these lacking electron density maps. These structural changes were indirectly confirmed by two spectroscopic experiments using circular dichroism (CD) (Figure 3B) and fluorescence intensity detection (Figure 3C). CD and fluorescence intensity detection can be used to detect changes in secondary structures and side chains [35].

In order to observe changes in the secondary structure, CD was measured at 4 °C and 25 °C. As a result, the overall shape of CD spectra was maintained according to the temperature; however, the Y axis value decreased. This implies that changes in secondary structure were temperature-dependent. In a similar way, when residues with a ring structure, such as tryptophan and phenylalanine, were detected at 4 °C and 25 °C using a fluorescence spectrophotometer, fluorescence absorbance decreased as temperature increased, which suggests changes in residues with a ring structure. The dynamicity of the residues increases while the number of detectable residues by fluorescence spectrophotometry decreases. Changes in temperature led to conformational changes in the protein structure, including the sites related to the physiological properties of IN [36].

### 2.3. Structural Properties of HIV-1 Integrase Catalytic Core Region

**Figure 4:**
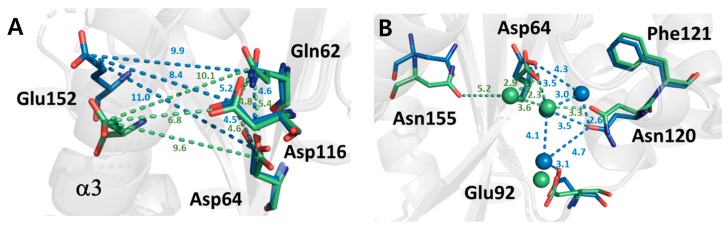
Comparison of the structures of the DDE triad (Asp, Asp, and Glu) and its vicinities. (**A**) Superimposed DDE triad region non-cryogenic (green) and cryogenic (blue) structures of HIV-1 integrase catalytic core domain. (**B**) Hydrogen bond network mediated by water molecules near the active site.

The catalytic core region of IN-CCD is composed of a triad formed by Asp64, Asp116, and Glu152. The DDE triad plays an important role in the activity of IN and is conserved in all types of retrovirus integrases. Compared to the DDE triad of the non-cryogenic and cryogenic structures, Asp116 in the loop region undergoes almost no structural changes. However, in the case of Glu152 in helix III, it shows a significant conformational change from the backbone to the side chain, as a change in the orientation of helix III. Asp64 shows a change of side chain, whereby it is shifted by 33.5 degrees, indicating that the change of Asp64 took place to maintain the DDE Triad since Glu152 moved downward. This series of changes in the active site has resulted in a change in the electrostatic network between the DDE triad and Gln62. As the temperature changes from cryogenic to ambient temperature, the distance between residues increases, such that the volume of the active cavity increases (Figure 4A).

At the bottom of the active site, several residues, including Asp64 and Asn120, are connected via water molecules (Figure 4B). This region is related to interaction with tDNA when forming the STC intasome and structural differences were found between the cryogenic and non-cryogenic structures here [23]. At ambient temperature, the number of water molecules making up the hydrogen bond network was reduced from 3 to 1, and the direction of the sidechains, such as Asp64, Glu92, and Asn120, also changed. As the number of charged residues captured by water molecules decreased, it can be assumed that the residues capable of electrostatic interaction with tDNA increased. In fact, there was no trapping force by the water molecules, such that the B-factor of the related residues in the non-cryogenic structure was increased (~6 Å^2^). Our results indicate that the conformational flexibility of the active site and its vicinity is improved at the non-cryogenic structure compared to the cryogenic structure.

### 2.4. Protein Dynamics of IN-CCD by SFX-XFELs

While the absolute B-factor may be affected by errors associated with the experimental conditions, in general, its distribution indicates the relative static and dynamic flexibility of the protein in crystal form [37]. Because both the structures were obtained from the same sample, we analyzed the B-factor distribution to study the effects of temperature on the thermal dynamics of IN-CCD. Fundamentally, the basal B-factor of the structure determined using XFEL was higher than the basal B-factor of the structure determined using Synchrotron. Because of this discrepancy, it was difficult to directly analyze the protein dynamics using B-factor alone. In order to solve these problems, the B-factor of the cryogenic structure was subtracted from the B-factor of the non-cryogenic structure, and the difference value was plotted and analyzed. As a result, the B-factors of all the molecules, the B-factor of the backbone main chain, and the B-factor of the side chain were obtained. In the case of the backbone atom, the intensity of the electron density map was strong and clear, so data deviation is low. However, the data of the side chain atom and all the atoms were relatively less clear because the side chain is more dynamic than the main chain and the intensity of the electron density map for the side chain is relatively weak. By analyzing the B-factors calculated from three different atom groups (all atomic, only main chain, and only side chain residues), it was possible to determine which factors led to changes in the protein dynamics for all atom, main chain, and side chain residues. 

**Figure 5:**
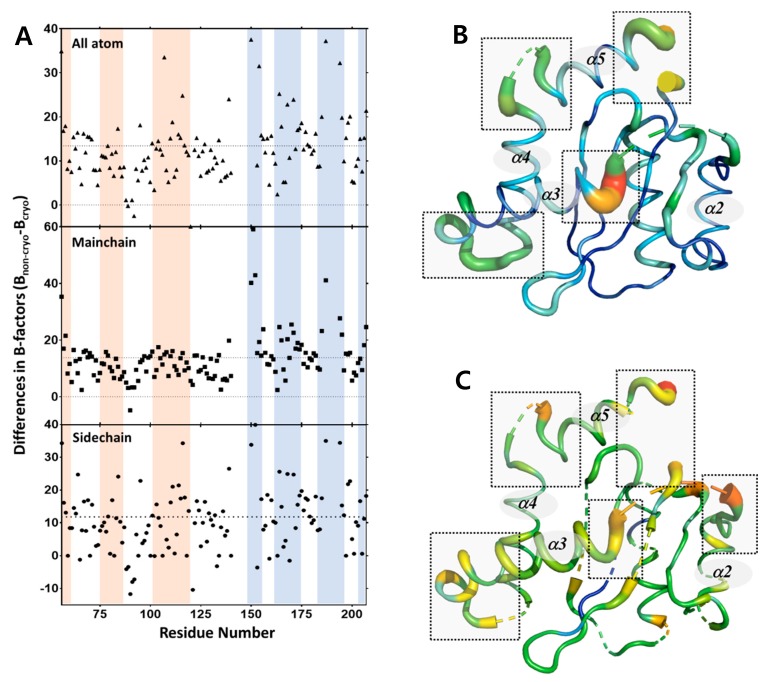
Differences in B-factors between non-cryogenic and cryogenic structures together with NMR data. (**A**) Difference in the B-factors (B_non-cryo_-B_cryo_) for all atoms, main chain atoms, and side chain atoms are plotted versus residue number. (**B**) Putty representation of the HIV-1 integrase catalytic core domain shows the differences in B-factors (B_non-cryo_-B_cryo_) of main chain atoms. (**C**) Putty representation of the HIV-1 integrase catalytic core domain shows the average values of the NMR dynamics results (R_1_, R_2_, ^15^N -{^1^H} NOE). Note that a greater width and the color red indicates a higher value, while a narrower width and the color blue denotes a lower value.

From the perspective of all atom residues, the distribution of the different B-factors highlights a more rigid region in comparison to a more flexible region, with more pronounced B-factor deviations observed in the non-cryogenic structure (Figure 5A). Helix III, helix IV, and the loop between helix IV and helix V show much larger deviations in B-factors (16–53 Å^2^) between the two structures compared to the average B-factor difference of 11 Å^2^. The average B-factor of the DDE triad in the non-cryogenic structure (94.5 Å^2^) was 22.2 Å^2^ higher than that in the cryogenic structure (72.3 Å^2^), and the B-factor of the DDE triad was 10.5 Å^2^ higher than that of the entire structure. This is consistent with a previous study that the DDE triad had larger thermal motions at higher temperatures than other regions. However, this must take into account the possible effects of Bragg termination during the XFEL pulse [38,39]. In addition, the residues in the range 167–174, which is the LEDGF IBD binding site, also had a high difference in the B-factor, with a difference of 19.2 Å^2^ compared to the average value of whole protein. The regions that undergo temperature-dependent conformational changes may play a role in the function of the protein. 

To further define the dynamics of the IN-CCD under physiological conditions, we analyzed our heteronuclear ^15^N- {^1^ H} NOE values and previously published ^15^N R_1_, R_2_ values [33]. These values denote the flexibility of the IN-CCD on a pico-nanosecond timescale and are useful for determining the parts of the protein that are disordered. Interestingly, the results of the dynamics analysis using NMR versus those using B-factor differences were very well-correlated. The helix III region of the 140–153 residues near the active site were found to be highly dynamic. Similarly, highly dynamic structures were also observed at the C-termini of residues 185–195. The residues that make up the DDE triad were also found to be highly dynamic (Figure 5B–C). Thus, the overall structural dynamics of these regions, as seen in the crystal data, are consistent with the backbone dynamics observed in solution NMR.

The dynamics analysis shows that the data from crystal structures can be compared to the dynamic information from solution NMR. In results, the Pearson correlation coefficient between B-factor difference and NMR dynamic data is 0.4, confirming positive correlation. Thus, SFX-XFELs allows access to the non-cryogenic structure, which is a more accurate representation of the structural information of IN-CCD in its native state. The use of SFX to accurately determine the structural details of a protein at ambient temperature may represent an important contribution to improving our understanding of structure-function relationships. In other words, structural analysis using SFX-XFELs can provide an important insight into the relationship between the structure and function of proteins.

## 3. Discussion

In this study, IN-CCD was crystallized at a high density for SFX-XFELs experiments via the optimization of crystallization conditions. Monoolein (9.9 MAG) was used as a carrier matrix [40]. The mixing ratio of 9.9 MAG and protein crystals was optimized to produce a stable flow stream. To our knowledge, this study represents the first non-cryogenic structure of IN-CCD and its dynamic properties using SFX-XFELs.

The data presented in this study confirm several previous findings on HIV-1 integrase and reveal new information on the existence of structural changes that are temperature-dependent. The comparison between the cryogenic and non-cryogenic structures was direct because the crystallization conditions for SFX-XFELs and conventional crystallography were identical. Moreover, non-cryogenic structure did not suffer from cryogenic cooling or radiation damage. In this respect, the non-cryogenic structure determined at ambient temperature was helpful to improve our understanding of protein functions at a temperature optimum for their activity. Even though the unit cell volume of the non-cryogenic structure was greater (by approximately 3.35%) than that of the cryogenic structure due to the cryogenic cooling effect, we directly compared both structures, and found significant structural changes associated with protein functions and characteristics.

By overlapping the cryogenic structure with the non-cryogenic structure, we observed a good agreement with RMSD 0.31 Å, except for helix III and rotamer changes in the protein residues. The topology of helix III in HIV-1 IN-CCD in the non-cryogenic structure was similar to that in the HIV-1 IN-CCD bound with DNA (Figure S2). Therefore, the free state of IN-CCD has a similar structural feature to the HIV-1 IN-CCD complexed with DNA and exhibits high dynamic mobility. It is also considered that the dynamicity of HIV-1 IN-CCD decreases when it is combined with DNA (Figure S3). The B-factor values of the DDE triad of several HIV-1 IN-CCD compared with the cryogenic and non-cryogenic structures show a consistent result, supporting our conclusion (Figure S4). In addition, we noted a difference in the number and distribution of crystalized water molecules. These structural changes were experimentally confirmed by comparing electron density maps. The structural changes observed in our results suggested temperature-dependent protein dynamics. At ambient temperatures, the non-cryogenic structure had a relatively wider catalytic cavity volume in the DDE triad compared to the cryogenic structure. Moreover, the residues involved in binding to LEDGF IBD, vDNA, and tDNA also underwent structural changes. Even though our results provide proof of the protein-protein interactions of IN-CCD, further insights could be obtained by carrying out biochemical experiments using techniques such as isothermal titration calorimetry and surface plasmon resonance. 

The distribution of B-factors highlights the flexibility of regions including the helices and C-terminal in comparison to the overall structures, with smaller B-factor deviations observed in the non-cryogenic structure. Helix III, helix IV, the loop between helix IV and helix V, and part of the C-terminus showed much greater deviations in B-factors compared to the average difference. These regions of the protein structure were involved in interactions with the binding partner or active site, such that their mobility could affect the kinetics of catalytic reactions and interactions with cellular binding partners. The ^15^N NMR dynamics analysis presented here confirms that the results of the dynamic analysis using crystal data correlated well with those obtained from solution NMR. Therefore, our results suggest that the non-cryogenic structure obtained using SFX-XFELs better represents the native protein in solution. 

In conclusion, this study contributes to the understanding of molecular dynamics and original topology which are related to the function of IN-CCD protein through the analysis of total structure difference by temperature. This result suggests that XFEL may expand its application to more comprehensive structural areas including protein dynamics.

## 4. Materials and Methods

### 4.1. Protein Purification and Crystallization

HIV-1 IN-CCD was expressed and purified following the previously described methods [20] Briefly, TRX-hexa-his tagged IN-CCD was expressed in *E. coli* BL21 (DE3) cells, and the cells were lysed by sonication. IN-CCD was then purified using Ni- immobilized metal affinity chromatography and size-exclusion chromatography. The purified protein was concentrated to 15–20 mg/mL. ^15^N-labeled IN-CCD was expressed in M9 media with ^15^N-labeled ammonium chloride. The purification scheme was the same as that of unlabeled IN-CCD.

All crystallization experiments were carried out in 1-mL tubes. The tubes are filled with 1:1 ratio mixture of protein and crystallization solution using the previously described conditions, with slight modifications, to obtain a high density of crystals (0.2 M ammonium sulfate, 0.1 M sodium cacodylate trihydrate pH 6.5, 30% *w/v* PEG 8000) [20]. The tubes were sealed tightly using parafilm and stored in an incubator at 16 °C for 1–2 weeks.

### 4.2. Data collection and Processing at PAL 

The cryogenic crystal data were collected using a beamline 5C of Pohang Light Source-II, in a 100 K liquid nitrogen stream with an X-ray beam at a wavelength of 0.983 Å. Diffraction data was processed using HKL-2000 (HKL Research, Inc., Charlottesville, VA, USA). The cryogenic crystal presented a trigonal space group P 31 2 1 with one monomer in an asymmetric unit. 

The SFX data of the non-cryogenic structure were collected at the Nano Crystallography and Coherence Imaging (NCI) experimental station of PAL-XFEL [41,42]. The IN-CCD crystals in tube were centrifuged and the precipitant solution was discarded. Approximately 30 μL of 9.9 MAG was added to the tube. The mixture of crystals and 9.9 MAG was then homogenized using a 100 μL Hamilton gas tight syringe. The sample was transferred into a lipidic cubic phase (LCP) injector through a syringe containing sample with LCP loading adaptor. The sample in the LCP injector was delivered to the X-ray interaction point with a 100-μm diameter nozzle at an average flow rate of 2 nL/min in the MICOSS (multifarious injection chamber for molecular structure study) [43]. Data were collected at a 30-Hz repetition rate using X-ray pulses have a pink beam spectrum centered at 9.7 keV (λ = 1.3 Å). The photon flux was (1–2)  ×  10^11^ photons per pulse within a duration of 20 fs. The incident X-ray beam was focused at 5  ×  5 μm^2^ (FWHM) using Kirkpatrick-Baez mirrors [44]. The diffraction images were collected using a MX225-HS (Rayonix, LLC) detector with a 4  ×  4 binning mode (pixel size: 156 μm × 156 μm) (Figure S1). The crystal hit rate was between 7–20%. The real-time diffraction status was monitored online by OnDA [45]. The collected data were processed using ClickX [46], Cheetah and CrystFEL [47,48]. The indexing rate was approximately 60–75%. The crystals belonged to the *P 31 2 1* space group with cell parameters similar to cryogenic crystals.

### 4.3. Structure Determination and Refinement

For the non-cryogenic structure determination, the phase problem was solved via molecular replacement with Phaser-MR using the previously solved structure (PDB code: 1ITG) as a search model. Iterative model building was performed using Coot, and the structure refinement was carried out using Refmac and PHENIX.refine [49,50]. The structures were further refined, resulting in R_work_/R_free_ = 0.1766/0.2117 over a resolution range of 24.67–2.5 Å. Likewise, the cryogenic structure was refined, resulting in R_work_/R_free_ = 0.1994/0.2499 over a resolution range of 26.05–2.15 Å. The Ramachandran statistics of the final cryogenic/non-cryogenic structure had 99.26% in the preferred and 0.74% in the allowed regions with 0% outliers. All structural images were generated using PyMOL (https://pymol.org/). All structures were subsequently deposited in the Protein Data Bank (Appendix A).

### 4.4. Circular Dichroism and Fluorescence Spectroscopy

Using purified protein, buffer exchange was conducted using a PD-10 column (GE Healthcare, Chicago, IL, USA) with 20 mM sodium phosphate, 100 mM NaCl, and 1 mM BME. Far-UV CD spectra were monitored using a JASCO J-815 spectrometer (Jasco, Easton, MD, USA). The path length was 2 mm, and the instrument parameters were set to a sensitivity of ~30 millidegrees, a response time of 1 s, and a scan speed of 100 nm/min. The spectra were recorded as the average of 3 scans [51].

To measure the fluorescence intensity, steady-state fluorescence measurements were carried out using a PerkinElmer LS 55 fluorescence spectrometer (PerkinElmer, Waltham, MA, USA) The slit widths for excitation and emissions were set at 10 nm and 10 nm, respectively. Samples were excited at 280 nm. Emission spectra were recorded from 270 to 450 nm [52].

### 4.5. ^15^N-{^1^H} NOE Experiment

The steady-state heteronuclear ^15^N-{^1^H} NOE data were collected for 2048(t2) × 384(t1) dimensions with a 3-second recycle delay using ^15^N-labeled IN-CCD. ^15^N-{^1^H} NOE spectra were acquired by interleaving pulse sequences and then processed for analysis [53,54]. 

### 4.6. DPI Analysis for Non-Cryogenic and Cryogenic Structures

The final refined structures were run through the online Diffraction Precision Index (DPI) server [55]. DPI was calculated for residues highlighted in Figure 2. The positional shift in experimental data is measured using COOT.

## 5. Conclusions

In this study, we report the non-cryogenic structure data of IN-CCD, clearly representing its native structural properties. Our structural analysis results clearly show the difference between non-cryogenic and cryogenic structures. Especially, we could observe changes of hydrogen bond network mediated by water molecules and structural change in residues around helix III and DDE triad, which are related to the activity of integrase. This structural analysis is very informative to understand their functional activity in more detail. The B-factor in the non-cryogenic structure of IN-CCD also shows good agreement for their dynamics properties in solution state analyzed by NMR, which is not seen in the cryogenic structure. Several biochemical experiments such as CD and fluorescence spectrophotometer experiments have reaffirmed the structural change of the IN-CCD with temperature. To the best of our knowledge, this is the first report of non-cryogenic structure and dynamics analysis of IN-CCD combined with SFX-XFELs and NMR. The experimental method used herein may have application in the structural dynamics study of enzymes and MISC for imaging enzyme-catalyzed reactions. 

## Figures and Tables

**Table 1:** Data collection and refinement statistics.

	Non-Cryogenic Structure of IN-CCD	Cryogenic Structure of IN-CCD
**Data collection**		
Temperature	293 K	100 K
Space group	*P 31 2 1*	*P 31 2 1*
Cell dimension		
a, b, c (Å)	73.275 73.275 66.71	72.08 72.08 65.192
α, β, γ (°)	90.00 90.00 20.00	90.00 90.00 120.00
Number of collected images	330,698	N.A
Number of hits	40,024	N.A
Number of indexed patterns	27,311	N.A
Number of merged patterns	27,308	N.A
Indexing rate (%)	68.2	N.A
Resolution (Å)	24.67–2.5 (2.61–2.5) *	36.05–2.15 (2.25–2.15) *
R_merge_ (%)	N.A	2.89 (22.2)
R_split_ (%)	9.80 (77.37)	N.A
R_pim_ (%)	N.A	2.89 (22.2)
I/σ (I)	7.26 (1.45)	33.7 (4.4)
Completeness (%)	99.85 (100)	99.98 (100)
Multiplicity	406.99 (275.68)	1.9 (1.9)
CC_1/2_ (%)	99.35 (55.06)	99.9 (86.1)
**Refinement**		
Resolution (Å)	24.67–2.50	36.05–2.15
Number of reflections	7450	10,935
R_work_/R_free_	0.1766/0.2117	0.1994/0.2499
Cruickshank DPI (Å)	0.205	0.174
Number of atoms		
Protein	1098	1106
Water	12	29
B-factor (Å^2^)		
Wilson B/Overall B	57.46/67.36	41.39/54.02
Root mean square deviations		
Bond lengths	0.009	0.009
Bond angles	0.47	0.60
PDB code	6JCG	6JCF

* Values in parentheses are for highest-resolution shell.

## Supplementary Materials

Supplementary materials can be found at https://www.mdpi.com/1422-0067/20/8/1943/s1.; Table S1: DPI analysis for non-cryogenic and cryogenic structures of IN-CCD. Figure S1: Diffraction images of IN-CCD collected in PAL-XFEL using MX225-HS. Figure S2: Superimposed images of helix III of HIV-1 integrase/DNA complex (5U1C) together with non-cryogenic and cryogenic IN-CCD structure. Figure S3: Putty presentation of HIV-1 integrase catalytic core domain structure bound and non-bound with DNA. Figure S4: The B-factor comparison of the DDE triad in the non-cryogenic, cryogenic and other known structures of HIV-1 IN-CCD.

## Appendix A

The coordinates and structural factors have been deposited in the Protein Data Bank under the accession codes 6JCG (Non-cryogenic structure derived from SFX-XFELs) and 6JCF (Cryogenic structure derived from synchrotron). The raw diffraction data from PAL-XFEL is accessible in the Coherent X-ray Imaging Data Bank (CXIDB, http://cxidb.org/).
