# Supplementary material for: Non-Cryogenic Structure and Dynamics of HIV-1 Integrase Catalytic Core Domain by X-ray Free-Electron Lasers
Source: Int J Mol Sci. 2019 Apr 20;20(8):1943. doi: 10.3390/ijms20081943 (PMC6514806; doi:10.3390/ijms20081943)
Supplement: Supplementary file 1 [file ijms-20-01943-s001.pdf]

Non-cryogenic structure and dynamics of HIV-1 integrase catalytic core domain by X-ray free-electron lasers

Jae-Hyun Park [1†], Ji-Hye Yun [1†], Yingchen Shi [2,3], Jeongmin Han [1], Xuanxuan Li [2,3], Zeyu Jin [1], Taehee Kim [1], Jaehyun Park [4], Sehan Park [4], Haiguang Liu [2,*] and Weontae Lee [1,*]

[1] Structural Biochemistry & Molecular Biophysics Laboratory, Department of Biochemistry, College of Life Science & Biotechnology, Yonsei University, Seoul, Republic of Korea; wtlee@yonsei.ac.kr
[2] Complex Systems Division, Beijing Computational Science Research Center, Beijing, China 100193;
[3] Department of Engineering Physics, Tsinghua University, Beijing, China 100084;
[4] Pohang Accelerator Laboratory, Pohang, Gyeongbuk, Republic of Korea; jaehyun.park@postech.ac.kr
* Correspondence: hgliu@csrc.ac.cn (H.L.) and wtlee@yonsei.ac.kr (W.L.)
† These authors contributed equally.

Supplementary Materials

Table S1. DPI analysis for non-cryogenic and cryogenic structures of IN-CCD.

Figure S1. Diffraction images of IN-CCD collected in PAL-XFEL using MX225-HS.

Figure S2. Superimposed images of helix III of HIV-1 integrase/DNA complex (5U1C) together with non-cryogenic and cryogenic IN-CCD structure.

Figure S3. Putty presentation of HIV-1 integrase catalytic core domain structure bound and non-bound with DNA.

Figure S4. The B-factor comparison of the DDE triad in the non-cryogenic, cryogenic and other known structures of HIV-1 IN-CCD.

Table S1. DPI analysis for non-cryogenic and cryogenic structures of IN-CCD.

Residue	DPI of non-cryogenic structure (Å)	DPI of cryogenic structure (Å)	The positional shift in experimental data (Å)/(°)
Glu168	0.25(0.26)	0.21(0.228)	1.05(CD) ,15.21
Glu170	0.27(0.285)	0.21(0.201)	4.53(CD), 71.72
Tyr194	0.26(0.294)	0.21(0.253)	1.72(OH), 14.72
Arg187	0.27(0.291)	0.21(0.243)	1.26(NH1)
Arg199	0.22(0.272)	0.18(0.199)	0.77(NH1)
Tyr83	0.17(0.163)	0.14(0.161)	0.77(OH), 7.82
Lys185	0.20(0.197)	0.15(0.181)	2.25(NZ)
His114	0.20(0.216)	0.16(0.163)	0.66(CE1), 6.4
Phe121	0.18(0.177)	0.18(0.199)	0.57(CZ) *
Asn155	0.25(0.248)	0.21(0.219)	1.53(ND2) *
Asp64	0.20(0.223)	0.16(0.171)	0.83(OD2), 18.85
Asn120	0.18(0.187)	0.18(0.191)	1.71(OD1) *
Tyr99	0.18(0.201)	0.15(0.173)	0.54(OH), 4.57
Glu87	0.20(0.211)	0.18(0.204)	0.68(OE2), 9.19
Glu92	0.20(0.205)	0.19(0.203)	0.82(CD)
Glu96	0.19(0.199)	0.16(0.178)	0.52(OE1)
Gln95	0.22(0.263)	0.18(0.222)	0.63(CD) *

Values in parentheses are for specific atom used to measure positional shift.
Second values in 'The positional shift in experimental data' column are for angular change.
* The positional shift also appears in the backbone.

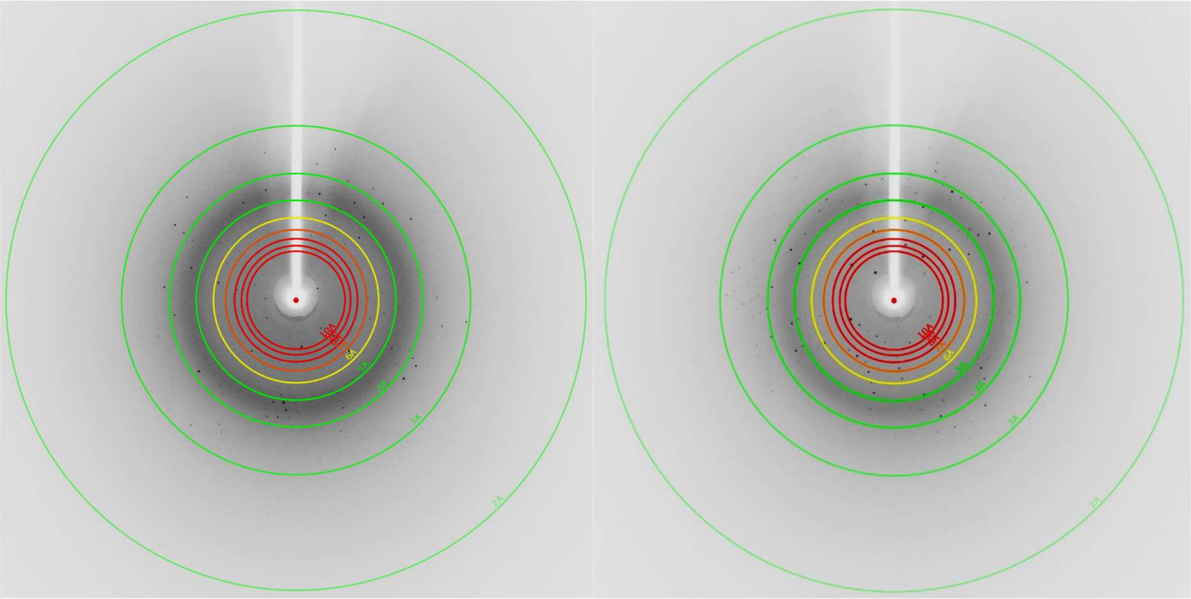

Figure S1. Diffraction images of IN-CCD collected in PAL-XFEL using MX225-HS. Diffraction spots from IN-CCD crystals extending to beyond 2.8Å resolution, with unit-cell parameter a=b=73.275 Å, c=66.71 Å, α=β=90°, γ=120°

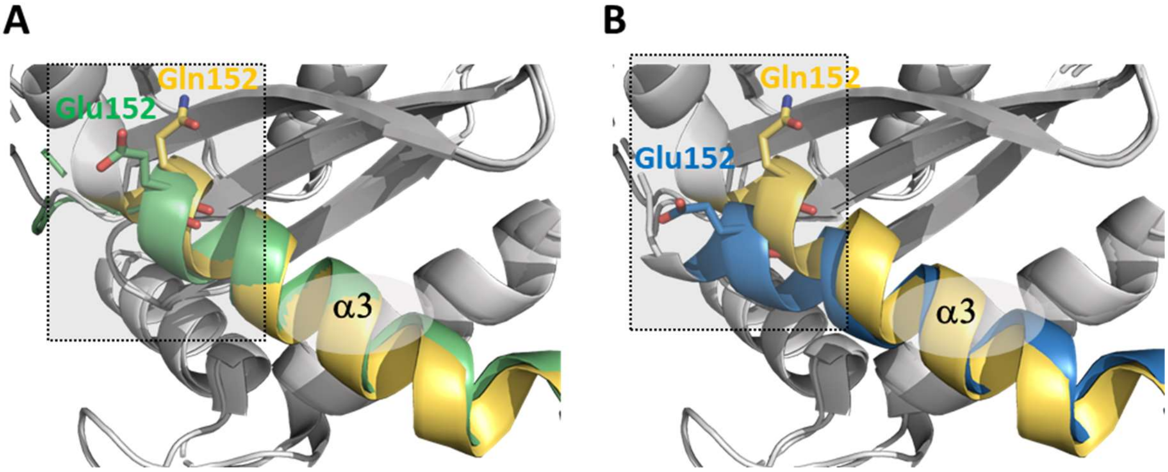

Figure S2. Superimposed images of helix III of HIV-1 integrase/DNA complex (5U1C) together with non-cryogenic and cryogenic IN-CCD structure. (**A**) Overlap representation of non-cryogenic (Green) and 5U1C (Yellow) structures. (**B**) Overlap representation of cryogenic (Blue) and 5U1C structures. 5U1C is a structure of HIV-1 integrase/DNA complex.

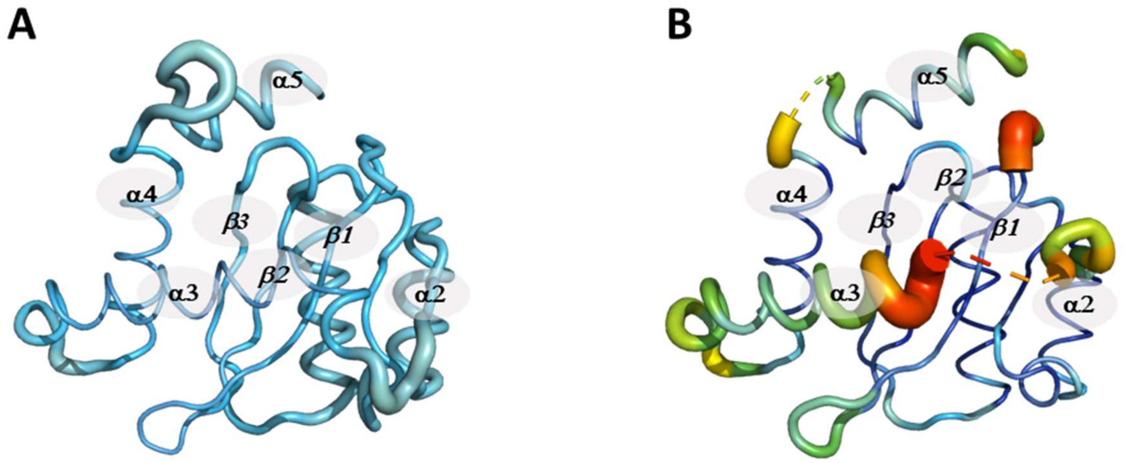

Figure S3. Putty presentation of HIV-1 integrase catalytic core domain structure bound and non-bound with DNA. (**A**) Atomic displacement parameter: color-coded B-factor distribution of the HIV-1 integrase catalytic core domain extracted from 5U1C. (**B**) Color-coded B-factor distribution of the HIV-1 integrase catalytic core domain of the non-cryogenic crystal structure.

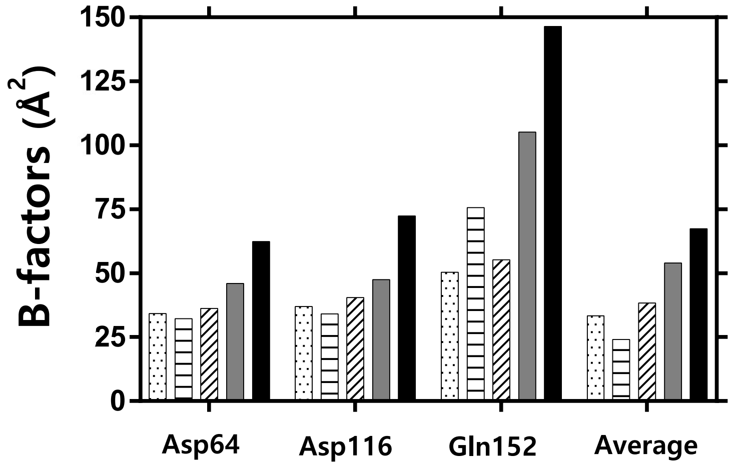

Figure S4. The B-factor comparison of the DDE triad in the non-cryogenic, cryogenic and other known structures of HIV-1 IN-CCD. The B-factor values of the DDE triad (Asp64, Asp116, and Gln152) and the average B-factors of all protein atom represented in a plot. (1BL3 (□); 1BIS (⊟); 1BI4 (◪); Cryogenic structure (■), and Non-cryogenic structure (■)).
